# Building Bridges: The Homemade Laparoscope Project for Medical Education

**DOI:** 10.1007/s40670-025-02388-7

**Published:** 2025-04-09

**Authors:** Manuel Cevallos, Samantha Dinh, Alicia C. Nguyen, Danielle Vildorf

**Affiliations:** 1 Medical Education Department, School of Medicine, Creighton University, 3100 N. Central Ave. Suite 706 K, Phoenix, AZ 85012 USA; 2 Surgery Department, College of Medicine, The University of Arizona, Phoenix, USA

**Keywords:** Medical education, Anatomy, Laparoscope, Abdomen, Medical students

## Abstract

We describe the construction of a homemade laparoscope that undergraduate and medical students can use to learn abdominal gross anatomy from a different perspective. Students can also explore basic concepts in the laparoscopic procedure. This reliable and low-cost instrument can reduce the learning gap in low-resource universities.

Abdominal anatomy is crucial in medical education, supporting clinical practice across specialties. Visceral topography assists in examination, diagnosis, trauma assessment, pathology, surgery, and radiological interpretation. Laparoscopy improves clinical outcomes, 3D orientation, and teamwork skills [1, 2] while promoting interest in surgery [2]. Additionally, 98.9% of students believe it enhances understanding and enthusiasm for anatomy [3]. However, the high cost of laparoscopic towers (€56,250 +) restricts broader adoption [4].

Researchers at Creighton University (Phoenix) developed a $200 homemade laparoscope to overcome cost barriers, enabling abdominal cavity evaluation in cadavers with or without formaldehyde preservation. The homemade laparoscope was presented at the American Association for Anatomy’s annual meeting in Washington, D.C., on March 25–27, 2023.

The homemade laparoscope consists of the following (Fig. 1A): (1) The laparoscopic system: the endoscope and light source, DEPSTECH WiFi Borescope, 5.0 MP HD wireless endoscope, Semi-Rigid, 16-inch focal distance, Snake Inspection Camera with 2200 mAh battery, 2592 × 1944 resolution HD inspection camera, 8.5-mm probe, with software for smartphones or tablets featuring image orientation, video/image capture. (2) Gas source: It uses air from the room. Dual LED Digital Pressure Gauge Air Compressor Pump regulates pressure. (3) Laparoscopic instruments: Veress needle (VN), two grasper forceps, and two Trocars (insert and cannula). (4) Other: rigid plastic 10-mm diameter tubing, scalpel, blade, hemostats, and gloves. (5) Body donors can be preserved with or without formaldehyde.

**Fig. 1:**
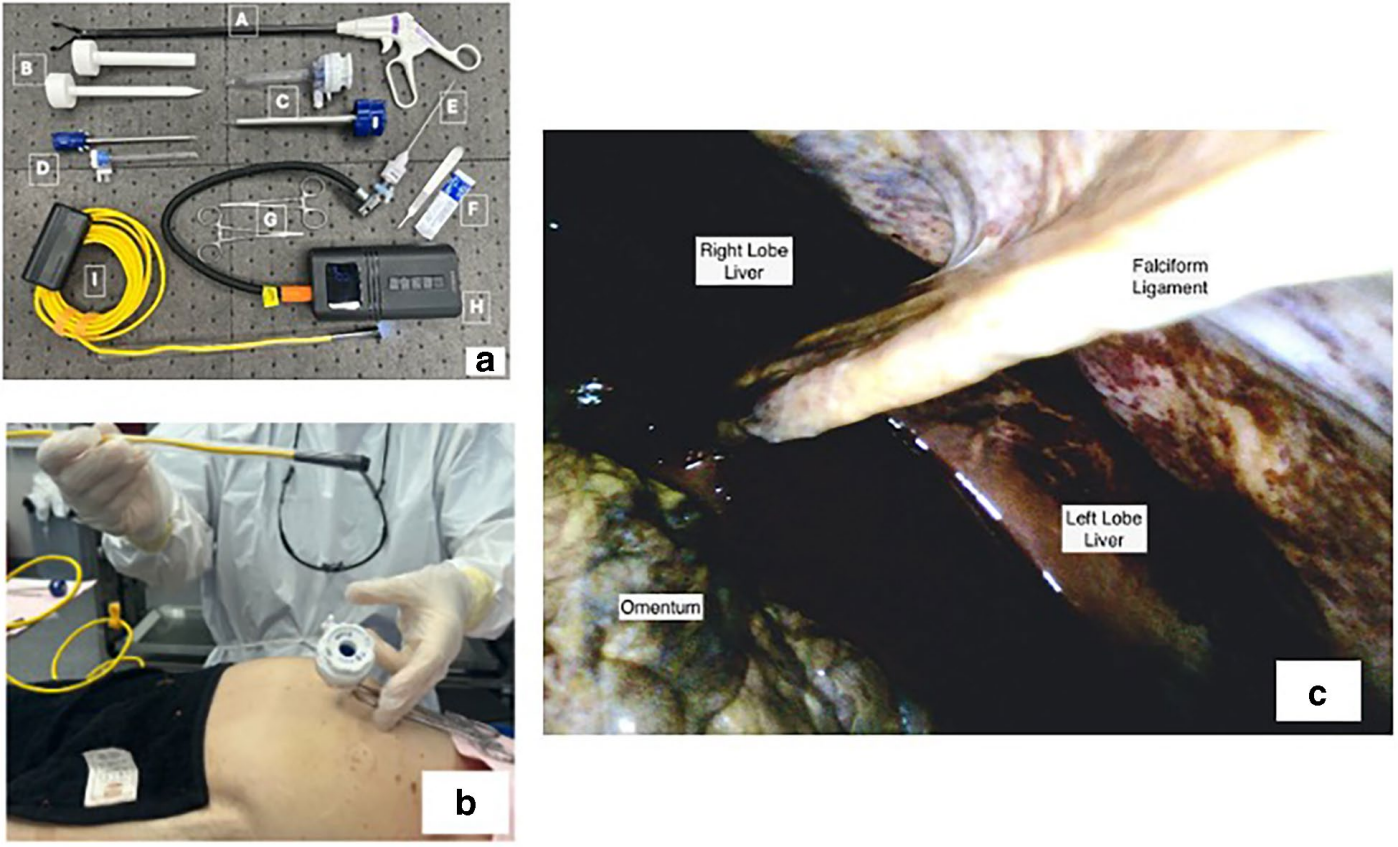
Homemade laparoscope. **a** (**A**) Grasper forceps. (**B**) 3D printer trocar. (**C**) 10-mm trocar and insert. (**D**) 5-mm trocar and insert. (**E**) Veress needle. (**F**) Scalpel and blade. (**G**) Hemostats. (**H**) LED Digital Pressure Gauge Air Compressor Pump. (**I**) DEPSTECH WiFi Borescope. **b** Laparoscope previous insertion. **c** The abdominal cavity shows the right and left hepatic lobe, falciform ligament, and omentum

First, download the Depstech software from the Apple or Google store on a screen device. Turn on and pair the Depstech device. Mirror it or connect to a monitor via HDMI for observation or image/video capture. Then, the “Depstech” endoscope is introduced into the 10 mm, rigid, transparent tube (cannula) at the tip and fixed. The classic standard procedure for performing a laparoscopy includes the following steps: (1) Make a 0.5-cm-longitudinal incision at the superior middle border of the umbilicus. Clamp both sides and pull up to create a tent. Introduce the Veress needle until it reaches the abdominal cavity, using the Linea alba as a guide. (2) When the needle tip is in the abdominal cavity, connect the Pressure Gauge Air Compressor Pump and turn it on to create the pneumoperitoneum. In cadavers with soft embalming, the intraabdominal pressure will be around 6–8 mmHg. With high formaldehyde concentration, the pressure may reach 25–30 mmHg. For higher pressures, increase progressively in pulses of 5 mmHg (on/off the device) and slow flow to achieve controlled and uniform abdominal distention. (3) After producing the pneumoperitoneum, quickly remove the VN and insert the trocar through the same opening, enlarging the incision if needed. For commercial trocars with an air valve, reconnect the Pressure Gauge Air Compressor Pump and resupply air as necessary. For 3D printed trocars without an air valve, reinsert the VN at the side of the abdomen to maintain the pneumoperitoneum. (4) With a stable pneumoperitoneum, remove the insert from the trocar and quickly insert the homemade laparoscope previously assembled (Fig. 1B). Make a fast visualization of the abdomen, then direct the laparoscope to the abdominal wall, identifying a point between the left middle clavicular line and the inferior border of the ribs. Medial to this point, insert a second trocar. This technique also identifies a good point for reinserting the VN if necessary. (5) Identify suitable points on the abdominal wall and introduce additional trocars and forceps; forceps and laparoscope can be changed in position to have a better vision to explore.

The baroscope and air pump were purchased online. The VN and laparoscopic forceps were obtained from the hospital’s surgical laparoscope representative or through a laparoscope company’s educational program. Trocars and forceps can also be 3D printed.

Hands-on anatomy courses enhance learning, engagement, and leadership among medical and undergraduate students. They also improve visual-spatial awareness, attention to detail, critical thinking, communication, and teamwork. Laparoscopy on preserved cadavers facilitates an understanding of abdominal anatomy (Fig. 1C) and decision-making, although it is expensive. We offer a cost-effective alternative for exploring abdominal anatomy using any donor type, increasing accessibility for students from various institutions.
